# A Simple Ultraperformance Liquid Chromatography-Tandem Mass Spectrometry Method for Measurement of Cortisol Level in Human Saliva

**DOI:** 10.1155/2019/4909352

**Published:** 2019-03-03

**Authors:** Syed N. Alvi, Muhammad M. Hammami

**Affiliations:** Clinical Studies and Empirical Ethics Department, King Faisal Specialist Hospital & Research Center, MBC-03, P.O. Box 3354, Riyadh 11211, Saudi Arabia

## Abstract

A simple ultraperformance liquid chromatography-tandem mass spectrometry assay for measurement of cortisol level in human saliva was developed and validated. Saliva samples containing cortisol were spiked with tolperisone as internal standard (IS) and extracted with a mixture of methyl tert-butyl ether and hexane (8:2, v:v). After solvent evaporation, residue was reconstituted in 100 *μ*l mobile phase. Analysis was performed on Atlantis dC18 column (2.1 × 100 mm, 3 *μ*m particle size) with a mobile phase composed of acetonitrile and 2 mM ammonium acetate (50:50, v:v) and delivered at a flow rate of 0.3 ml/minute. Mass spectrometry acquisition was performed with multiple reaction monitoring in positive-ion mode for cortisol and IS (m/z: 363.1 → 121.0 and 246.0 → 97.9, respectively). Retention times of cortisol and IS were about 1.35 and 2.45 minutes, respectively. The relationship between cortisol level and peak area ratio of cortisol to IS was linear in the range of 0.5-100 ng/ml. Intra- and interday coefficient of variation and bias were ≤ 9.0% and ≤12.0%, respectively. Mean extraction recoveries of cortisol and IS from saliva samples were 92% and 94%, respectively. Using the method, cortisol was found to be ≥ 86% stable in processed (24 hours at room temperature or 48 hours at -20°C) and ≥ 91% stable in unprocessed (24 hours at room temperature or 20 weeks at -20°C) saliva samples. Further, the method was successfully applied to determine daily cortisol profile in saliva samples of a healthy volunteer.

## 1. Introduction

Cortisol is a steroid hormone that plays an important role in regulating a wide range of physiological and pathological processes that involves immune response, electrolyte balance, blood pressure, and metabolism among others [1, 2]. Measurement of cortisol level in biological fluids is used in the diagnosis of diseases related to adrenal, pituitary, and hypothalamic function, including Cushing's syndrome and Addison's disease [3, 4].

Measurement of cortisol level in saliva samples is particularly attractive as it reflects biologically active cortisol and samples can be obtained stress-free [5, 6]. Several studies assessed cortisol level in saliva, using radioimmunoassay (RIA) [7, 8], enzyme immunoassay (EIA) [9–11], high-performance liquid chromatography (HPLC) [12, 13], or liquid chromatography-tandem mass spectrometry (LC-MS/MS) [14–17]. Salivary cortisol levels measured by RIA are strongly correlated with levels measured by a highly sensitivity EIA (r = 0.98, P < 0.001) [11] and LC-MS/MS (r = 0.99, P < 0.01) [15]. However, although immunoassays have high sensitivity, they often suffer from low selectivity due to cross-reactivity with related substances [18]. On the other hand most of the reported LC-MS/MS assays are based on solid-phase extraction [15, 17] and/or the use of deuterium-labeled cortisol as an internal standard (IS) [14, 16], which may not be feasible in some clinical laboratories.

Here we describe a simple, precise, and rapid ultraperformance liquid chromatography-tandem mass spectrometry (UPLC-MS/MS) assay for determination of cortisol level in human saliva using tolperisone as an internal standard (IS). The method was validated according to US FDA guidelines [19] and used to determine cortisol stability in saliva under various clinical laboratory conditions and applied to monitor the level of cortisol in saliva samples collected from healthy volunteers.

## 2. Experimental

### 2.1. Chemicals and Reagents

All chemicals were of analytical grade unless stated otherwise. Hydrocortisone (cortisol) and tolperisone were purchased from Acros organic, NJ, USA, and Sigma-Aldrich, MO, USA, respectively. Ammonium acetate, methyl tert-butyl ether, hexane, and acetonitrile (HPLC grade) were purchased from Fisher Scientific, NJ, USA. HPLC grade water was prepared by reverse osmosis and further purified by passing through a Synergy Water Purification System (Millipore, Bedford, MA, USA). The study was approved by the Research Ethics Committee of King Faisal Specialist Hospital & Research Centre, Riyadh, Saudi Arabia, under Research Advisory Council (RAC# 2160008).

### 2.2. Instrument and Chromatographic Conditions

The liquid chromatograph tandem mass spectrometer (LC-MS/MS) consists of Xevo-TQD detector equipped with Z-spray, an atmospheric pressure ionization (API) interface, Acquity UPLC H-Class system, integrated solvent, and sample manager (Waters Corporation, Milford, MA, USA). Analysis was performed at room temperature using a reversed phase Atlantis dC18 column (2.1 × 100 mm, 3 *μ*m particle size), steel column protected by a column guard in-line filter (2 mm, 0.2 *μ*m). The mobile phase was composed of 2 mM ammonium acetate and acetonitrile (50:50, v:v). It was filtered using (47 mm, 0.2 *μ*m pore size) Supor membrane disc filter (Pall Gelman Laboratory, MI, USA) and delivered at a flow rate of 0.3 ml/minute. The electrospray ionization (ESI) source was operated in the positive-ion mode at a capillary voltage of 1.5 kV and cone voltage of 36 V. Nitrogen was used as the nebulizing and desolvation gas at a flow rate of 1000 L/hr. Argon was used as the collision gas maintaining cell pressure at 3.6 E^−003^ mbar. An optimum collision energy of 20 eV was applied for both cortisol and IS. The ion source and the desolvation temperatures were maintained at 150°C and 500°C, respectively. Cortisol and IS were detected and quantified in the positive-ion mode; product ion response was measured in multireaction monitoring (MRM) mode at set transitions mass to charge (m/z) of 363.1 → 121.0 and 246.0 → 97.9, respectively. Mass lynx Ver 4.1 (Waters Corporation, Milford, MA, USA) software working under Microsoft Window XP professional environment was used to control the instrument parameters, data acquisition, peak integration, peak smoothing, and signal-to-noise ratio measurements.

### 2.3. Preparation of Standard and Control Samples

Cortisol and IS stock solutions were prepared in methanol (1.0 *μ*g/ml). Nine calibration standards in the range of 0.5-100 ng/ml and four quality control concentrations (0.5, 1.5, 50, and 90 ng/ml) were prepared in normal human saliva. IS working solution (20 ng/ml) was prepared in water. Aliquots (1.0 ml) of saliva samples were transferred into 7 ml glass culture tubes and stored at -20°C until used.

### 2.4. Saliva Samples for Calibration Standards and Controls

Unstimulated saliva samples were collected from healthy volunteers by direct spitting in sterile (110 mm × 28 mm dia) Corning 50 ml centrifuge tubes (Sigma-Aldrich, USA) and stored at -20°C until analyzed.

### 2.5. Preparation of Samples

50 *μ*l of the IS working solution (20 ng/ml) was added to each 1.0 ml unknown saliva sample, calibration standard, or quality control samples in 7 mL glass culture tubes and vortexed for 30 seconds. 4.0 ml mixture of methyl tert-butyl ether and hexane (8:2, v:v) was added, vortexed for two minutes, and centrifuged at 4000 rpm for 15 minutes at 20°C. The supernatant clear layer was transferred to a clean borosilicate culture tube and dried under gentle steam of nitrogen at 40°C. The residue was reconstituted in 100 *μ*l mobile phase, 2 mM ammonium acetate and acetonitrile (50:50, v:v) and 10 *μ*l of the clear solution was injected into the LC-MS/MS system.

### 2.6. Extraction Recovery

Extraction recovery of cortisol was measured by comparing cortisol peak areas in two samples. In one sample blank saliva was spiked with cortisol and then extracted. In the second sample, blank saliva was extracted first and then spiked with the same amount of cortisol. This was done in four replicates at four concentrations (0.5, 1.5, 50, and 90 ng/ml). Recovery of the IS was determined in the same fashion at a concentration of 20 ng/ml. Extraction recovery was calculated as mean cortisol (or IS) peak area in spiked-before-extraction samples divided by mean cortisol (or IS) peak area in spiked-after-extraction times 100.

### 2.7. Stability Studies

Two QC samples concentrations (1.5 and 90 ng/ml) in saliva were used for stability studies. Five aliquots of each concentration were extracted and immediately analyzed (baseline). Five aliquots of each concentration were allowed to stand on the bench-top for 24 hours at room temperature; five aliquots were stored at -20°C for 20 weeks, before being processed and analyzed; and five aliquots were processed and stored at room temperature for 24 hours or at -20°C for 48 hours before analysis. Fifteen aliquots of each concentration were stored at −20°C for 24 hours. They were then left to completely thaw unassisted at room temperature. Five aliquots were analyzed and the rest stored at to -20°C for another 24 hours. The cycle was repeated three times.

### 2.8. Calculations

In order to correct for endogenous level of cortisol in “blank” saliva, we used the difference in peak area ratios between each consecutive concentration as the response (rather than the peak area ratio) [20]. The difference in peak area ratio was plotted against the concentration. Bias (%) was calculated as the difference between measured and nominal concentration divided by nominal concentration times 100, whereas coefficient of variation (%) was calculated as standard deviation divided by mean concentration times 100.

### 2.9. Matrix Effect

Matrix effect was evaluated by comparing the peak areas of extracted blank saliva that was then spiked with cortisol at four concentrations (0.5, 1.5, 50, and 90 ng/ml) and IS (20 ng/ml) with the corresponding peak areas obtained by direct injection of standard solutions prepared in mobile phase.

### 2.10. Method Validation

The method was validated (specificity, recovery, linearity, accuracy, precision, and stability) according to the US Food and Drug Administration (FDA) bioanalytical method validation guidance [19].

### 2.11. Saliva Samples from a Healthy Volunteer

About 4.0 ml saliva samples were collected within 20-30 minutes by direct spitting in sterile Corning 50 ml centrifuge tubes. Samples were collected starting at 4:30, 10:00, 15:30, 19:30, and 22:00 hours on a rest day and on a work day and stored at -20°C until analyzed.

## 3. Results

### 3.1. Separation and Quantification

**Figure 1:**
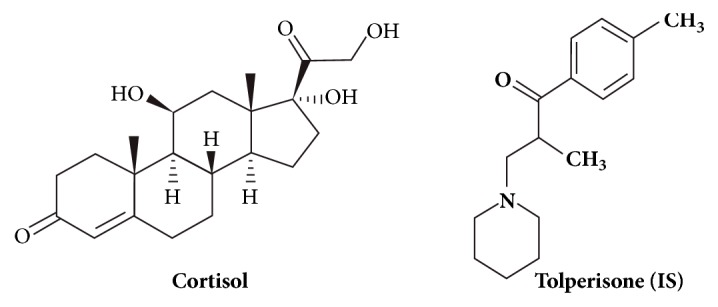
Chemical structures of cortisol and tolperisone (IS).

**Figure 2:**
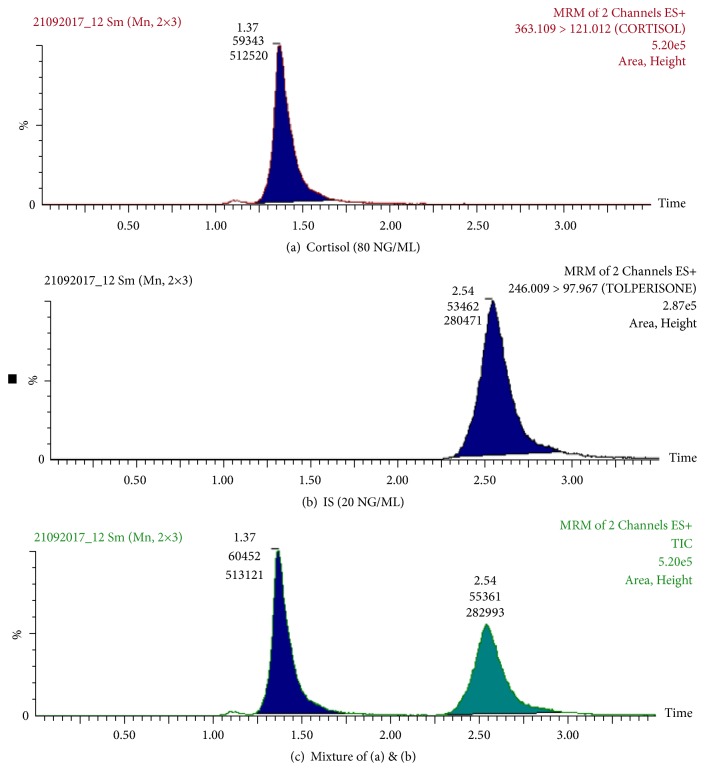
Total ion current and multiple reaction monitoring chromatograms of cortisol and the internal standard (IS) tolperisone in human saliva.

Figure 1 depicts the chemical structures of cortisol and tolperisone (IS). Liquid chromatographic (LC) conditions were optimized using a mobile phase composed of 2 mM ammonium acetate and acetonitrile (50:50, v:v) at a flow rate 0.3 ml/minute. The relatively high proportion of acetonitrile facilitated column low back pressure and shorter run time (< 3.0 minutes). The product and precursor ions were determined by infusing a standard mixture of cortisol and tolperisone (1.0 *μ*g/ml in methanol) in the mass spectrometer using a configured software program (IntelliStart, obtained from Waters Corporation, Milford, MA, USA). Cortisol and IS each produced two product ions peaks: 363.1 → 121.0 or 363.1 → 97 and 246.0 → 97.9 or 246.0 → 69.9, respectively. Transition 363.1 → 121.0 for cortisol and transition 246.0 → 97.9 for IS were chosen to quantitate cortisol level, since they gave the best response.  Figure 2 depicts total ion current (TIC) and MRM chromatograms of cortisol and IS.

### 3.2. Matrix Effect

Matrix effect is common in atmospheric pressure ionization LC-MS/MS analysis. It is mainly due to interference of molecules originating from the sample matrix that coelute with the compound(s) of interest during the process of ionization, causing ionization suppression or enhancement. Matrix effect, usually up to ±15%, is considered as insignificant [21, 22]. Mean matrix effect was an ion suppression of -9.7% and -13.7% for cortisol and IS, respectively.

### 3.3. Specificity

**Figure 3:**
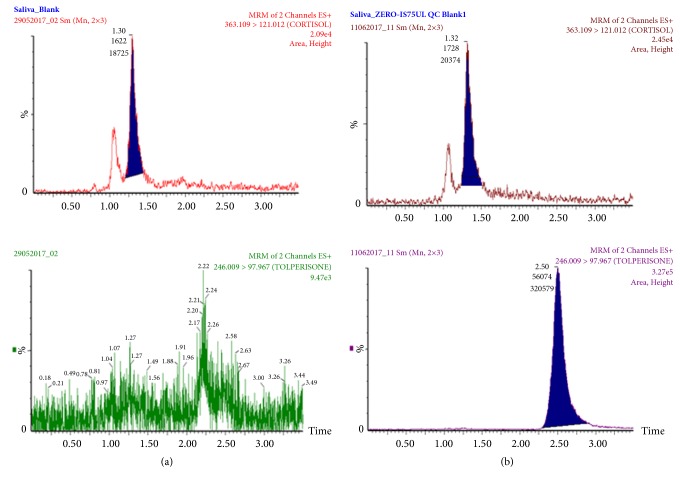
Multiple reaction monitoring chromatogram of blank human saliva used in preparation of standard and quality control sample. (a) Blank saliva, (b) blank saliva spiked with internal standard.

The specificity of the assay was determined by screening six different batches of blank human saliva, in addition to seven cortisol-related compounds (cortisone, progesterone, 17 *α*- hydroxy progesterone, prednisone, prednisolone, methyl prednisone, and testosterone). All solutions were 1.0 *μ*g/ml in methanol: water (1:1, v:v) and 10 *μ*l was injected into the system. No interference with the peaks of cortisol or the IS was obtained.  Figure 3 depicts representative chromatograms of human saliva that was used in preparation of calibration curve and quality control samples.

### 3.4. Recovery

The results of extraction recovery of cortisol and the IS are presented in Table 1. Mean extraction recoveries were 92% for cortisol and 94% for the IS.

### 3.5. Linearity and Limit of Detection and Quantification

**Figure 4:**
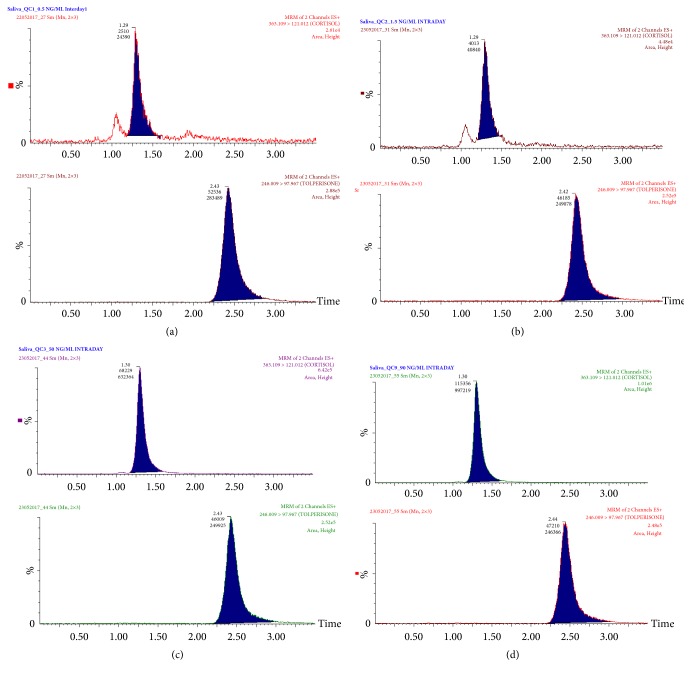
Multiple reaction monitoring chromatograms of four quality control samples ((a) 0.5, (b) 1.5, (c) 50, and (d) 90 ng/ml) spiked with 20 ng/ml internal standard (IS).

Linearity of the assay was evaluated by analyzing a series of cortisol standards at nine different concentrations over the range of 0.5–100 ng/ml in saliva. Corresponding peak area ratios and concentrations were subjected to regression analysis. Mean equation obtained from eight standard curves was y= 0.0188 (x) + 0.0117, with R^2^ (SD) = 0.9960 (0.0039). The detection and quantification limits were as 0.3 ng/ml and 0.5 ng/ml, respectively.  Figure 4 represents UPLC-MS/MS chromatograms of four QC samples (0.5, 1.5, 50, and 90 ng/ml) spiked with 20 ng/ml of IS.

### 3.6. Precision and Bias

The intraday and interday precision and bias (Table 2) were evaluated by analyzing four QC samples (0.5, 1.5, 50, and 90 ng/ml). The intraday precision and bias (n = 10) ranged from 2.4% to 9.0% and from –5.5% to 12.0%, respectively. The interday precision and bias were determined over three different days (n=20). They ranged from 3.9% to 8.4% and from –2.0% to 10.3%, respectively.

### 3.7. Stability

Cortisol and IS stability in processed and unprocessed saliva samples was investigated (Table 3). Cortisol in processed samples was found to be stable for 24 hours at room temperature (≥88%) and 48 hours at −20°C (86%). Cortisol in unprocessed samples was stable for at least 24 hours at room temperature (≥92%), 20 weeks at −20°C (≥91%), and after three freeze-thaw cycles (≥93%). Further, no significant change in chromatographic behavior of cortisol or the IS was observed under any of the above conditions.

### 3.8. Application of the Method

**Figure 5:**
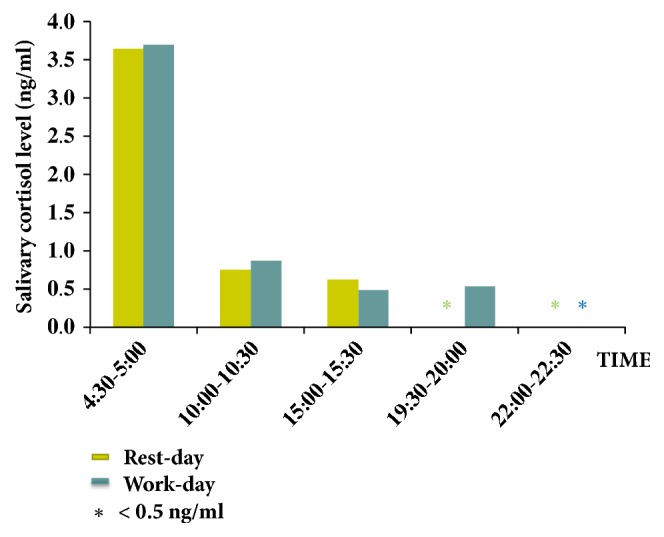
Level of cortisol in saliva samples collected from a healthy volunteer at different intervals on two different days.

The method was used to determine salivary cortisol profile in a healthy volunteer on two different days. The results are presented in Figure 5. As expected, cortisol levels were highest in the early morning period and declined to unmeasurable level at 10:00 to 10:30 PM.

## 4. Discussion

Measurement of cortisol level plays an important role in the diagnosis of adrenal dysfunction as well as in studying related physiological conditions [1–4]. A number of liquid chromatographic methods have been reported for cortisol determination in various biological matrixes [23, 24]. Measuring cortisol level in saliva has several advantages over other matrixes, including convenience in sample collection, avoidance of stress associated with vein puncture, and measuring biologically active cortisol level rather than total cortisol level [5].

Few LC-MS/MS-based assays have been reported for cortisol measurement in saliva [14–17]; they used solid-phase extraction and/or deuterium-labeled cortisol as IS. In general deuterium-labeled analytes have been used as IS in LC-MS/MS analysis, since they permit identical fragmentation of the IS and the assayed analyte. However, advancement in LC-MS/MS softwares allowed the successful use of non-deuterium-labeled ISs. We elected to use a chemically similar compound to cortisol, tolperisone, as an IS. Tolperisone gave major fragment peak response at collision energy of 16 eV compared to 20 eV for cortisol. Nevertheless, collision energy of 20 eV produced reliable and consistent results for the IS and thus was chosen.

We used a simple liquid-liquid extraction for sample preparation. Liquid-liquid extraction is considered a classic approach and is widely used in sample preparation for qualitative and quantitative analysis. Its main advantage is low cost, as it does not require expensive equipment compared to solid-phase extraction.

Cortisol level in saliva ranges from 0.6 to 15 ng/ml (1.5-40 nmol/L) when measured by immunoassay [7]. Although immunoassays are highly sensitive, they often suffer from cross-reactivity with cortisol-related compounds. In fact, cortisol levels obtained by immunoassays are 2.7 times higher than those obtained by LC-MS/MS [16]. Using the current assay, we found that cortisol level was about 3.5 ng/ml in the early morning and declined to unmeasurable level in the evening, in agreement with previous report [25].

## 5. Conclusion

The described UPLC-MS/MS method is simple, precise, and accurate for rapid measurement of cortisol level using 1.0 ml human saliva. The method uses readily available internal standard and was successfully used to determine cortisol stability under various laboratory conditions. Further, it was successfully applied to determine cortisol level in saliva samples obtained from a healthy volunteer.

## Figures and Tables

**Table 1:** Extraction recovery of cortisol and internal standard (IS) from saliva.

**Nominal concentration** **(ng/ml)**	**∗** **Spiked-before-extraction**		**∗** **Spiked-after-extraction**		**†Recovery** **(**%**)**
**Cortisol**	**Mean**	**SD**	**Mean**	**SD**	
0.5	973	76	1029	15	95
1.5	1652	131	1910	178	86
50	49728	4814	53252	4358	93
90	109762	9774	115761	4087	95
IS 20	103554	15923	109630	17990	94

*∗* represents mean peak area of 4 replicates and † mean peak area in spiked-before-extraction samples divided by mean peak area in spiked-after-extraction samples x100. SD: standard deviation.

**Table 2:** Intra-and inter-run precision and accuracy of cortisol assay.

**Nominal level** **(ng/ml)**	**Intra-day (n=10)**			**Inter-day (n=20)**		
	**Mean (SD)** **measured** **level**	**CV** **(**%**)**	**Bias** **(**%**)**	**Mean (SD)** **measured** **level**	**CV** **(**%**)**	**Bias** **(**%**)**
0.5	0.52 (0.05)	9.0	3.2	0.51 (0.04)	8.4	2.2
1.5	1.42 (0.09)	6.0	-5.5	1.47 (0.11)	7.7	-2.0
50	52.77 (1.49)	2.8	5.1	53.27 (2.61)	4.9	6.5
90	100.79 (2.38)	2.4	12.0	99.23 (3.86)	3.9	10.3

SD: standard deviation. CV: coefficient of variation = standard deviation divided by mean measured concentration x 100. Bias = measured level - nominal level divided by nominal level x 100.

**Table 3:** Stability of cortisol in human saliva.

**Stability **	**Storage condition**	**Spiked** **concentration (ng/ml)**	**Measured concentration (ng/ml)**	**SD**	**Stability (**%**)**
**Processed samples**	24 hr. (RT)	1.5	1.34	0.06	89
		90	78.91	3.39	88
	48 hr. (-20°C)	1.5	1.29	0.02	86
		90	77.48	1.61	86
**Un-processed samples**	24 hr. (RT)	1.5	1.59	0.04	100
		90	82.68	1.33	92
	20 wks (−20°C)	1.5	1.36	0.17	91
		90	87.90	4.73	98
**Freeze and Thaw samples**	FT: Cycle-1	1.5	1.42	0.07	95
	(−20°C)	90	85.33	2.28	95
	FT: Cycle-2	1.5	1.54	0.12	100
	(−20°C)	90	83.25	1.75	93
	FT: Cycle-3	1.5	1.43	0.11	95
	(−20°C)	90	86.39	2.76	96

RT: room temperature, hr.: hours, wks: weeks, FT: Freeze-Thaw. Stability (%): mean measured concentration (n=5) at the indicated time divided by mean measured concentration (n=5) at baseline x 100.

## Data Availability

The data used to support the findings of this study are available from the corresponding author upon request.
